# Wing Patterns in the Mist

**DOI:** 10.1371/journal.pgen.1000822

**Published:** 2010-02-05

**Authors:** Arnaud Martin, Durrell D. Kapan, Lawrence E. Gilbert

**Affiliations:** 1 Ecology and Evolutionary Biology, School of Biological Sciences, University of California Irvine, Irvine, California, United States of America; 2 Center for Conservation and Research Training, Pacific Biosciences Research Center, University of Hawaii at Manoa, Honolulu, Hawaii, United States of America; 3 Section of Integrative Biology, School of Biological Sciences and Brackenridge Field Laboratory, University of Texas at Austin, Austin, Texas, United States of America; University of Arizona, United States of America

The aesthetic appeal of butterfly wing patterns has been costly to their status as a tool of fundamental scientific inquiry. Thus, while mimetic convergence in wing patterns between edible “Batesian” mimics and distasteful models, or between different distasteful “Müllerian” mimics (species that cooperate to educate predators) has long been the subject of genetic analysis [1] and field experiments [2], most biology text books confine mimicry to sections on striking adaptations without applying these examples to broader topics of evolution. Meanwhile, the study of color patterns in animals, often tucked into the same sections of texts, is undergoing a revolution in this age of evo-devo and genomics [3]. Among insect models for studying color pattern, the genus *Heliconius* is gaining the attention of an ever-widening audience ([4]–[6]; Figure 1).

**Figure 1:**
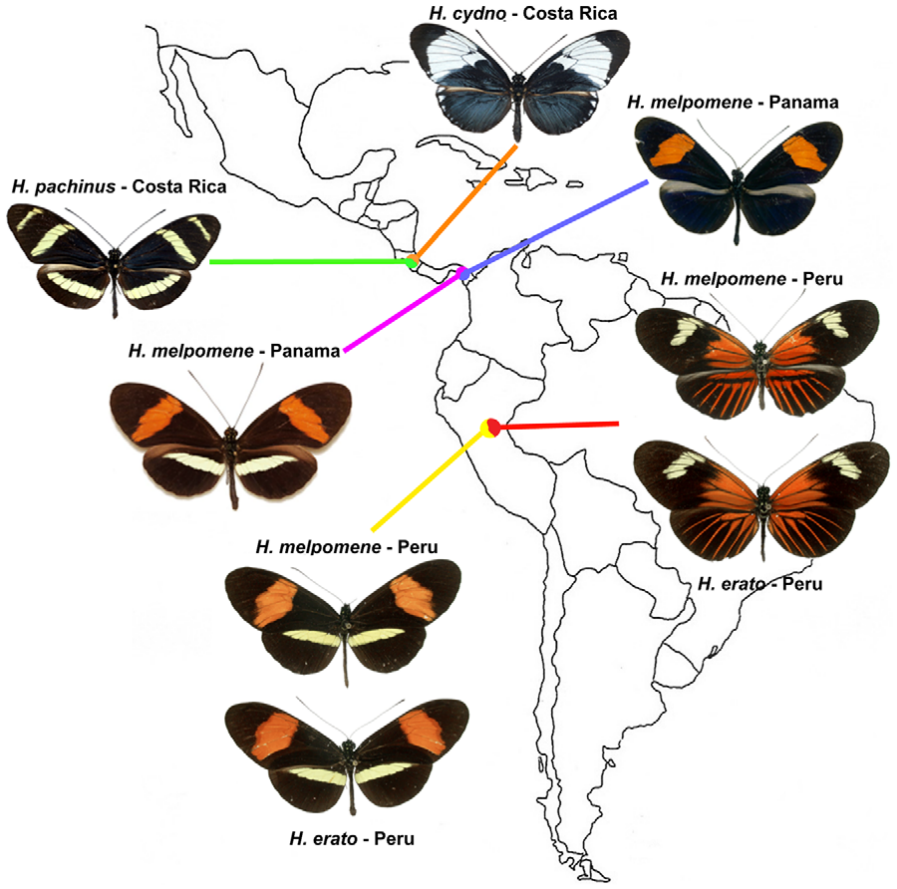
Three natural hybrid zones between parapatric populations of different *Heliconius* species. Only a subset of the phenotypic diversity and geographical distribution of the *erato*, *melpomene*, and *cydno* clades is represented. Müllerian mimicry is here illustrated by the convergence in wing patterns of *H. melpomene* and *H. erato* on both sides of the Peruvian Andes (yellow and red). *H. pachinus* and *H. cydno* are sister species that occasionally hybridize.

## *Heliconius*: Taxonomic Hotspot

Early in the 20th century, Oxford's pre-eminent evolutionist and student of insect color patterns, Edward B. Poulton, urged Harry Eltringham to study taxonomic relationships of a spectacularly colorful, mimetic, and diverse set of specimens pouring into European museums from field collectors across the Neotropics. Eltringham [7] distinguished *Heliconius erato* and *Heliconius melpomene* groupings and noted repeated mimetic convergence between them. However, within those groupings, he failed to distinguish species, races, and hybrids. In the mid 1950s, William Beebe and associates initiated studies of life history, behavior, systematics, and genetics of *Heliconius* at Simla in Trinidad. There, Michael Emsley elucidated biogeographic details of the system [8]. It soon became clear that many rare “taxa” described as species by museum workers were in fact recombinants occurring in narrow hybrid zones between two distinct mimetic races. In these zones Müllerian partners *erato* and *melpomene* each generate similar arrays of hybrid phenotypes, many of which would be sufficiently distinct to warrant separate species status when viewed out of context.

## Genetics of Parallel Mimetic Radiations

In the 1960s, genetic studies of *H. erato* and *H. melpomene* at Simla established the framework of classification of pattern loci in general use today [9]. In 1979, Turner [10] reported a strong discrepancy in levels of differentiation in color pattern versus allozyme loci across the geographical range of *erato* and *melpomene*. Thus, if viewed only through the lens of structural genes not manifest in the visible phenotype, few of the many races described for these species would be delimited. Later research in Peru on selection and gene flow in parallel interracial hybrid zones by James Mallet [11] set the stage for work on genomic hot spots described in this issue [12],[13].

Several teams have been busy in recent years trying to relate underlying allelic variation in color pattern observed in laboratory crosses and in natural hybrid zones to changes occurring in the genome. Classic genetic mapping previously showed that these adaptive polymorphisms in four different radiations were linked to homologous intervals [14]–[16]. In particular, the *B/D* locus, which controls the presence/absence of red patterns, and the *Yb/Cr* locus, which controls the presence/absence of a yellow bar, respectively map to homologous linkage groups between the co-mimics *H. melpomene* and *H. erato*, although co-mimetic phenotypes evolved independently. In other words, convergent evolution in wing patterning between species involved the same genetic intervals, and, since synteny between distantly related Lepidoptera is conserved [17], by extension, likely many of the same genes. This ignited a push to narrow the search to actual genes or nucleotide changes responsible for parallel wing pattern shifts, to illuminate genetic and developmental mechanisms responsible for generating spectacular and adaptive morphological diversity. Are *cis-*regulatory or *trans-*regulatory changes responsible for these polymorphisms [18],[19]? Do similar phenotypes reflect identical nucleotide changes, or independent functional changes in homologous genes or developmental pathways? The current work appears to be on a path that will help resolve questions about genotype phenotype connections.

## Hybrid Zones Uncover the Smoking Guns of Selection

The *Heliconius* system forms a unique replicated natural experiment to study the genetics of adaptive traits: allowing comparison between parapatric races of different phenotypes, between geographically distant races of similar phenotypes, and finally, between different species (co-mimics) across parallel inter-racial hybrid zones. The papers in this issue exploit this system by seeking signatures of selection across previously identified genetic intervals *B/D* and *Yb/Cr* in hybrid zones where populations of different phenotypes are admixed. Indeed, in these species mimicry ring structure on both sides of a hybrid zone imposes a strong positive frequent-dependent selection favoring common wing patterns [2],[11]. This is expected to result in a peak of population differentiation at causative genetic loci, because pattern alleles from race A that introgress into race B should be quickly eliminated according to their altered visual effects on pattern (and vice-versa). Accordingly, both Baxter et al. [13] and Counterman et al. [12] found peaks of population differentiation within *H. melpomene* and *H. erato* wing pattern loci, whereas unlinked regions of the genome showed no deviation from neutrality. Also, both studies form a consistent set of observations at a finer genomic scale by looking for haplotypes statistically associated with a certain phenotype. They found a rapid decay in linkage disequilibrium in these species, yet they did not identify completely fixed differences that would pinpoint wing pattern genes with confidence. However, both studies implicated a kinesin-motor gene (*kinesin*) as a *B/D* candidate gene, since it was close to a hotspot of genotype-by-phenotype association and also showed a higher expression level correlating with red pattern phenotypes. Similarly, both studies identified a “parallel” peak of genotype-to-phenotype association between polymorphism in a Leucine-Rich Repeat gene (*LRR*) and the *Yb/Cr* phenotypes. Finally, although the two studies are somewhat complementary in their design, they do not always converge in their results. For instance, while Baxter et al. sampled three geographically distant pairs of admixing populations, Counterman et al. focused their geographical sampling on a narrow area with a sharp transition in wing phenotypes where numerous generations of recombination have had the opportunity to break down variation around causative switch genes. In this latter study (and to a lesser extent in Baxter et al.), several hotspots of pattern association were observed in addition to *kinesin* and *LRR*. This raises the possibility that loci involved in pattern variation in each zone of a wing consist of several functional sites, whether they are coding or regulatory changes. While puzzling at first sight, this observation is consistent with the notion that these loci are supergenes with multiple wing patterning effects [14],[15], with the observation in *Drosophila* that tightly linked mutations of small effect participate in shaping an allele of major effect [20] and with a “Window/Shutter” model for interpreting variation in *Heliconius* wing patches and bands [21].

## The Best Model Organism for Integrative Biology?

Understanding the evolution of diversity will surely involve better integration of ecology, behavior, population genetics, and developmental biology, leading to new models of species diversification that incorporate well-characterized selective environments, adaptive peaks, and how networks of genes determine important phenotypes. *Heliconius* butterflies are clearly emerging as a premier model system for such integrative research (e.g., [22]). The studies reported here represent major steps forward in more respects than could be abstracted. But, to be honest, the genes that underlie *Heliconius* wing patterns still seem like a rainforest under a shroud of fog. Only a few canopy trees are visible as we fly over. It should be exciting when the clouds lift.
